# Improved gastrointestinal tolerance and iron status via probiotic use in iron deficiency anaemia patients initiating oral iron replacement: a randomised controlled trial

**DOI:** 10.1017/S0007114524002757

**Published:** 2024-11-28

**Authors:** Gokhan Koker, Yasin Sahinturk, Gulhan Ozcelik Koker, Muhammed Ali Coskuner, Merve Eren Durmus, Mehmet Mutlu Catli, Ayhan Hilmi Cekin

**Affiliations:** 1 Department of Internal Medicine, University of Health Sciences Antalya Training and Research Hospital, Antalya, Turkey; 2 Department of Gastroenterology, University of Health Sciences Antalya Training and Research Hospital, 07100 Muratpaşa, Antalya, Turkey

**Keywords:** Iron deficiency anaemia, Oral iron replacement, Probiotic supplementation, Lactobacillus plantarum 299v, Gastrointestinal tolerability, Iron status markers

## Abstract

This study aimed to investigate gastrointestinal tolerability, treatment persistence and iron status markers in patients with iron deficiency anaemia (IDA) who received oral iron replacement therapy (IRT) with *v*. without concomitant Lactobacillus plantarum 299v (*L. plantarum 299v*) probiotic supplementation. A total of 295 patents with newly diagnosed IDA were randomly assigned to receive either IRT alone (*n* 157, IRT-only group) or IRT plus *L. plantarum 299v* (*n* 138, IRT-Pro group) in this prospective randomised non-placebo-controlled study (ClinicalTrials.gov Identifier: NCT06521879). Gastrointestinal intolerance symptoms (at baseline, within the first 30 d of IRT and at any time during 3-month IRT), serum Hb levels (at baseline and 3rd month of IRT) and iron status markers (at baseline and 3rd month of IRT) were recorded. IRT-Pro group, when compared with IRT-only group, experienced significantly lower rates of gastrointestinal intolerance over the course of IRT (13·0 % *v*. 46·5 %, *P* < 0·001) and treatment discontinuation within the first 30 d (3·6 % *v*. 15·9 %, *P* < 0·001). At 3rd month of therapy, IRT-Pro *v*. IRT-only group had significantly higher serum levels for iron (76·0 (51·0–96·0) *v*. 60·0(43·0–70·0) µg/dl, *P* < 0·001) and transferrin saturation (20·1 (12·5–28·5) *v*. 14·5 (10·5–19·0) %, *P* < 0·001) and higher change from baseline Hb (0·9 (0·3–1·3) *v*. 0·4 (–0·1–1·1) g/dl, *P* < 0·001) levels. Use of *L. plantarum 299v* probiotic supplementation during the first 30 d of IRT in IDA patients significantly reduces the gastrointestinal burden of IRT (particularly abdominal pain and bloating), the likelihood of intolerance development (by ∼3 times) and treatment discontinuation (by∼5 times), as accompanied by improved serum Hb levels and serum iron markers.

Iron deficiency anaemia (IDA) is one of the most prevalent micronutrient deficiencies and a global health concern^(1,2)^. IDA has detrimental health consequences such as severe fatigue, dyspnoea and impaired thermoregulatory, neurocognitive and immune functions, in addition to its association with adverse outcomes in chronic kidney disease or chronic heart failure^(3–6)^.

Considering its hazard for the worldwide population, prevention and treatment of iron deficiency and IDA is of critical importance, while the strategies are mainly based on combination of dietary improvement, iron fortification of food, and iron supplementation^(7–9)^. Amongst these, oral iron supplementation (i.e. ferrous sulphate, gluconate and fumarate) is the most widely available and affordable method but its effectiveness is considerably limited by gastrointestinal side effects (in up to 70 % of patients), markedly impairing adherence to treatment and repletion of iron stores^(2,6,10–13)^.

Besides the inadequate iron intake, low iron bioavailability and absorption are also implicated in IDA pathogenesis and are highly affected by the gut microbiota composition^(8)^. The absorption of iron from diet or oral supplements is a complex mechanism, while oral iron supplements may also alter the composition of the gut microbiota towards a more pro-inflammatory milieu and decrease iron bioavailability^(6,8,14,15)^. Hence, strategies that consider enhancing iron absorption and reducing the risk of gastrointestinal side effects are important for effective iron replacement in patients with IDA^(6,16)^.

The gut microbiota enhances the host’s access to dietary iron by reducing the concentration of iron-binding compounds in the gut, and by converting Fe3+ to Fe2+, the absorbable ion form^(17)^. Due to the role of gut microbiota in regulating iron balance, probiotics have been suggested as a potential strategy to enhance iron absorption and alleviate deficiency, enabling a higher reduction of ferric iron to a bioavailable form, improved iron uptake by enterocytes, and an anti-inflammatory immune response^(6,8)^.

Use of probiotics, mostly the Lactobacillus and Bifidobacterium strains, as live microorganisms that improve composition of the gut microbiota, has gained public popularity because of their wide range of preventative and therapeutic potentials^(4,7,8,11)^.

Lactic acid-forming bacteria (lactobacilli) can increase iron absorption by lowering intestinal pH, activating phytases, causing shifts in gut microbiota metabolism and inducing anti-inflammatory immunomodulation^(6,16,18)^. This suggests that utilisation of probiotic bacteria may be a valuable clinical tool in prevention and amelioration of IDA, by optimising dietary iron bioavailability and thus improving iron status without the gastrointestinal burden of additional supplemental iron^(5–8)^. Specifically, the strain *Lactobacillus plantarum* 299v (*L. plantarum 299v*) with the ability to survive the passage through acid stomach and colonise the intestine^(16,19)^ has been shown to reduce bloating and abdominal pain in irritable bowel syndrome patients^(20,21)^ and to increase iron absorption and dietary iron bioavailability in IDA patients^(6,16,22,23)^. However, while probiotics were reported to be associated with amelioration of gastrointestinal intolerance symptoms in different settings^(20,21,24–27)^, their effects on gastrointestinal burden of iron replacement therapy (IRT) as well as on body iron status are less extensively studied in patients with IDA^(4,6–8,28,29)^.

Therefore, this study aimed to investigate the effects of *L. plantarum 299v* probiotic supplementation added to oral IRT on gastrointestinal burden, tolerability, treatment compliance and serum iron status markers in patients with newly diagnosed IDA.

## Materials and methods

### Study population

A total of 295 patients with newly diagnosed IDA who were planned to receive routine oral IRT were included in this prospective randomised non-placebo, controlled 3-month follow-up study (ClinicalTrials.gov Identifier: NCT06521879) conducted between September 2020 and March 2022 at a tertiary care internal medicine clinic. Patients were randomly assigned via simple randomisation method (computer-generated random number sequence) to receive either IRT alone (*n* 157, IRT-only group) or IRT plus *L. plantarum 299v* probiotic support (*n* 138, IRT-Pro group). Adult (aged > 18 years) treatment-naïve patients diagnosed with newly diagnosed IDA without previous IRT were included in the study, while those with irritable bowel syndrome, previous IRT therapy or intolerance to IRT and those with a known chronic disease (i.e. inflammatory bowel disease and celiac disease) or untreated active menometrorrhagia and haemorrhoid were excluded from the study.

Written informed consent was obtained from each subject following a detailed explanation of the objectives and protocol of the study which was conducted in accordance with the ethical principles stated in the ‘Declaration of Helsinki’ and approved by the Clinical Research and Ethics Committee of University of Health Sciences Antalya Training and Research Hospital (Date of Approval: 27/08/2020; Protocol No: 13/15).

### IDA definition

IDA was defined as having ferritin levels of < 20 ng/ml or transferrin saturation < 15 %, while the Hb levels were below 12 mg/dl^(30)^.

### Treatments

All patients received IRT with ferrous sulphate (Fe2^+^: 304·2 mg ferrous fumarate in pellet form, equivalent to 100 mg elemental iron) preparation (100 mg, once daily) for 3 months, while those in the IRT-Pro group also received daily (10B CFU) *L. plantarum 299v* (Probest®, Abdi Ibrahim, Turkey) probiotic supplementation for 30 d starting from the first day of IRT.

### Assessments

Data on gastrointestinal intolerance symptoms (loss of appetite, nausea, vomiting, abdominal pain, diarrhoea, constipation and bloating) were recorded at three time points including baseline, within the first 30 d of IRT and at any time during 3-month IRT. Overall, intolerance symptoms were evaluated based on new-onset (not present at baseline but appeared on IRT), ameliorated (present at baseline but disappeared on IRT) and total ((baseline + new onset) – (ameliorated)) symptom rates. A seven-item questionnaire was used to assess the presence of gastrointestinal intolerance symptoms during the past week on a binary scale (Yes or No), including the six items (nausea, vomiting, abdominal pain, bloating, constipation, diarrhoea) of Gastrointestinal Symptom Rating Scale^(31)^ and the loss of appetite (poor or very poor appetite) as the seventh item using the first question of the Appetite and Dietary Assessment Tool^(32)^.

Serum Hb levels (g/dl) and serum iron status markers including ferritin (ng/ml), iron (µg/dl), total iron-binding capacity (TIBC, µg/dl) and transferrin saturation (%) were recorded at baseline and at 3rd month of IRT. Samples for complete blood count were collected in K3EDTA tubes and analysed with an automated haematology analyser including Beckman-Coulter for Hb measurement and LISA 500 Plus automated chemical analyser (Hycell Diagnostics, Paris, France) for serum iron markers. Transferrin saturation was calculated by dividing serum iron by TIBC X 100.

Data on treatment discontinuation (persistence to IRT) were also recorded in study groups along with comparison of study variables in patients with *v*. without treatment discontinuation within the first 30 d of IRT.

### Statistical analysis

At least 189 patients were calculated to be included via sample size estimation (G * Power 3·1·9 program) based on a power of 80 % at a type I error of 0·05 and an effect size (w = 0·261) calculated using data from a previous study by Cekin et al.^(25)^. Given the high likelihood of missing data, a total of 200 patients were planned to be included in the study population with the use of 25 % lost to follow-up ratio.

Statistical analysis was made using IBM SPSS Statistics for Windows, Version 22.0 (IBM Corp.). Pearson’s chi-square test, Fisher’s exact test and McNemar test were used for analysis of categorical variables. Mann–Whitney U test was used for analysis of non-normally distributed numerical data while independent sample *t* test was used for normally distributed data. The number needed to treat (NNT) analysis was performed to determine how many patients must receive IRT-Pro instead of IRT to prevent one additional treatment discontinuation. Data are expressed as mean (sd, median, interquartile range, minimum-maximum and per cent (%) where appropriate. *P* < 0·05 was considered statistically significant.

## Results

### Patient demographics, intolerance development and treatment discontinuation

A total of 295 patents with newly diagnosed IDA were included in the study as randomly assigned to IRT-only (*n* 157) or IRT-Pro (*n* 138) groups. Mean (sd) patient age was 36·1(10·7) years and 96·3 % of patients were females. Both in the overall study population (*n* 295) and in patients with gastrointestinal intolerance symptoms (*n* 91), IRT-only and IRT-Pro groups were homogenous in terms of patient demographics (Table 1).

**Table 1.** Patient demographics, intolerance development and treatment discontinuation (Numbers and percentages; mean values and sd)

	Total (*n* 295)		IRT-only (*n* 157)		IRT-Pro (*n* 138)		
	*n*	%	*n*	%	*n*	%	*P* value
Patient demographics – overall							
Age (year)							
Mean	36·1		36·3		35·9		0·775
s d	10·7		10·5		10·9		
Gender, *n* (%)							
Male	11	3·7	8	5·1	3	2·2	0·186
Female	284	96·3	149	94·9	135	97·8	
Intolerance within 3 months IRT, *n* (%)							
Absent	204	69·2	84	53·5	120	87·0	< 0·001
Present	91	30·8	73	46·5	18	13·0	
Patient demographics – intolerance group (*n* 91)							
Age (year)							
Mean	37·2		37·4		36·9		0·755
sd	10·6		10·6		10·9		
Gender, *n* (%)							
Male	3	3·3	2	2·8	1	5·8	0·102
Female	88	96·7	71	97·2	17	94·2	
Treatment discontinuation, *n* (%)							
Total	36	12·2	28	17·8*	8	5·8	0·002
Within 30 d	30	10·2	25	15·9**	5	3·6	
Day of discontinuation							
Median	11		11		13		0·237
min-max	7–15		7–16		7–13		
Symptoms within 30 d in discontinuers (*n* 30), *n* (%)							
Loss of appetite	2	6·7	2	6·7	0	0·0	–
Nausea	7	23·3	5	16·7	2	6·7	
Vomiting	1	3·3	1	3·3	0	0·0	
Abdominal pain	6	20·0	5	16·7	1	3·3	
Diarrhoea	2	6·7	2	6·7	0	0·0	
Constipation	9	30·0	7	23·3	2	6·7	
Bloating	3	10·0	3	10·0	0	0·0	

IRT: Iron replacement therapy; IRT-only: received IRT alone; IRT-Pro: received IRT plus *L. plantarum 299v.*

Independent *t* test, Mann–Whitney U test, Pearson’s chi-square test, Fisher’s exact test.

*P < 0·01 and **P < 0·001 compared with IRT-Pro group.

Overall, 91 (30·8 %) of 295 patients reported gastrointestinal intolerance symptoms within 3 months of IRT. Patients in the IRT-Pro group compared with those in the IRT-only group had significantly lower rate of gastrointestinal intolerance development within 3 months of IRT (13·0 % *v*. 46·5 %, *P* < 0·001) (Table 1).

Treatment discontinuation within 3 months of IRT occurred in 36 (12·2 %) of 295 patients, while it was within the first 30 d of IRT in 30 (10·2 %) patients. Overall (17·8 *v*. 5·8 %, *P* < 0·01) and first 30-day (15·9 % *v*. 3·6 %, *P* < 0·001) treatment discontinuation rates were significantly higher in the IRT-only group than in the IRT-Pro group (Table 1).

IRT-Pro had an NNT of 3, indicating that 3 patients have to be treated with IRT-Pro instead of IRT-only to prevent one additional treatment discontinuation.

Among patients who discontinued IRT within the first 30 d, constipation was the leading symptom (30·0 %), followed by nausea (23·3 %) and abdominal pain (20·0 %), all of which were particularly noted in the IRT-only group (23·3 %, 16·7 % and 16·7 %, respectively) (Table 1).

### Intolerance data at baseline and within 3 months of iron replacement in study groups

Baseline rates for abdominal pain (17·4 *v*. 4·5 %, *P* < 0·001) and diarrhoea (5·8 *v*. 1·3 %, *P* = 0·049) were significantly higher in the IRT-Pro group (*n* 138) than in the IRT-only (*n* 157) group (Table 2).

**Table 2.** Intolerance symptoms at baseline and during 3 months of IRT in study groups (Numbers and percentages)

		IRT			IRT-Pro			
Intolerance symptoms		*n*	*n*	%	*n*	*n*	*n*	*P* value*
Loss of appetite								
Baseline		157	2	1·3	138	0		0·500
During IRT	New onset	132	8	6·1	133	1	0·8	**0·019**
	*Ameliorated*		*1*	*0·8*		*–*		
	Total		9	6·8		1	0·8	**0·010**
*P* value (baseline *v*. total)^†^			**0·008**			1·000		
Nausea								
Baseline		157	0		138	0		–
During IRT	New onset	132	23	17·4	133	5	3·8	**< 0·001**
	*Ameliorated*		–			–		
	Total		23	17·4		5	3·8	**< 0·001**
*P* value (baseline *v*. total)^†^			**< 0·001**			0·063		
Vomiting								
Baseline		157	0		138	0		
During IRT	New onset	132	3	2·3	133	0	0	0·122
	*Ameliorated*		**–**			–		
	Total		3	2·3		0	0·0	0·122
*P* value (baseline *v*. total)^†^			0·250			–		
Abdominal pain								
Baseline		157	7	4·5	138	24	17·4	**< 0·001**
During IRT	New onset	132	20	15·2	133	0	0	**< 0·001**
	*Ameliorated*		*1*	*0·8*		*20*	*15·0*	
	Total		26	19·7		4	3·0	**< 0·001**
*P* value (baseline *v*. total)^†^			**< 0·001**			**< 0·001**		
Diarrhoea								
Baseline		157	2	1·3	138	8	5·8	**0·049**
During IRT	New onset	132	5	3·8	133	4	3·0	0·749
	*Ameliorated*		**–**			*6*	*4·5*	
	Total		7	5·3		6	4·5	0·765
*P* value (baseline *v*. total)^†^			0·063			0·754		
Constipation								
Baseline		157	2	1·3	138	6	4·3	0·153
During IRT	New onset	132	18	13·6	133	6	4·5	**0·010**
	*Ameliorated*		*1*	*0·8*		*2*	*1·5*	
	Total		19	14·4		10	7·5	0·073
*P* value (baseline *v*. total)^†^			**< 0·001**			**0·031**		
Bloating								
Baseline		157	38	24·2	138	28	20·3	0·421
During IRT	New onset	132	9	6·8	133	7	5·2	0·585
	*Ameliorated*		*15*	*11·4*		*24*	*18·1*	
	Total		32	24·2		11	8·3	**< 0·001**
*P* value (baseline *v*. total)^†^			0·267			**0·006**		

IRT: Iron replacement therapy; IRT-only: received IRT alone; IRT-Pro: received IRT plus *L. plantarum 299v.*

New-onset: not present at baseline but appeared on IRT; ameliorated: present at baseline but disappeared on IRT; total: ((baseline + new onset) – (ameliorated)).

*Fisher exact test, Pearson’s chi-square test, ^†^McNemar test (*baseline v. during 3 months of IRT).*

All relevant values were formatted in italic and bold face.

In the IRT-only group, symptom rates significantly increased from baseline over the course of iron replacement (*n* 132), including loss of appetite (1·3 *v*. 6·8 %, *P* = 0·008), nausea (0·0 *v*. 17·4 %, *P* < 0·001), abdominal pain (4·5 *v*. 19·7 %, *P* < 0·001) and constipation (1·3 *v*. 14·4 %, *P* < 0·001). In the IRT-pro group (*n* 133), significant decrease from baseline rates was noted in the abdominal pain (17·4 *v*. 13·3 %, *P* < 0·001) and bloating (20·3 *v*. 8·3 %, *P* = 0·006), while constipation (4·3 *v*. 7·5 %) showed significant increase from baseline (Table 2).

Loss of appetite (0·8 *v*. 6·8 %, *P* = 0·010), nausea (3·8 *v*. 17·4 %, *P* < 0·001), abdominal pain (3·0 *v*. 19·7 %, *P* < 0·001) and bloating (8·3 *v*. 24·2 %, *P* < 0·001) were significantly less common in the IRT-Pro group than in the IRT-only group (Table 2).

Considering the intolerance symptoms newly emerged under IRT, the likelihood of developing de novo loss of appetite (6·1 *v*. 0·8 %, *P* = 0·019), nausea (17·4 *v*. 3·8 %, *P* < 0·001), abdominal pain (15·2 *v*. 0·0 %, *P* < 0·001) and constipation (13·6 *v*. 4·5 %, *P* = 0·010) were significantly higher in the IRT group than in the IRT-Pro group (Table 2).

### Serum iron status markers

At baseline, serum ferritin levels (5·0 (3·0–7·0) *v*. 7·0 (4·0–12·0) ng/ml, *P* < 0·001) and transferrin saturation (10·05 (5·2–16·0) *v*. 12·1 (8·1–17·1)%, *P* = 0·029) were significantly lower in the IRT-Pro group (*n* 138) than in the IRT-only (*n* 157) group (Table 3).

**Table 3.** Serum iron status markers from baseline to 3rd month of iron replacement therapy (Median values and interquartile ranges)

	Total			IRT			IRT-Pro			
Serum iron status	*n*	Median	IQR	*n*	Median	IQR	*n*	Median	IQR	*P* value
Ferritin (ng/ml)										
Baseline	295	6·0	4·0–9·0	157	7·0	4·0–12·0	138	5·0	3·0–7·0	**< 0·001**
3rd month	259	17·0	11·0–24·0	129	16·0	8–24	130	17·5	15–23	0·094
Change from baseline		11·0	2·0–17·0		5·0	–1·0–15·0		13·0	8·0–17·0	**< 0·001**
Iron (µg/dl)										
Baseline	295	45·0	28·0–64·0	157	47·0	34·0–64·0	138	39·0	24·0–64·0	0·087
3rd month	259	65·0	44·0–87·0	129	60·0	43·0–70·0	130	76·0	51·0–96·0	**< 0·001**
Change from baseline		15·0	–2·0–33·0		8·0	–6·0–23·0		23·5	5·0–48·0	**< 0·001**
TIBC (µg/dl)										
Baseline	295	403·0	374·0–444·0	157	406·0	38·01–438·0	138	403	374·0–446·0	0·869
3rd month	259	385·0	355–421·0	129	396·0	374·0–421·0	130	368·5	327·0–402·0	**< 0·001**
Change from baseline		–24·0	–60·0–11·0		–11·0	–40·0–24·0		–45·5	–76·0–(–3·0)	**< 0·001**
Transferrin saturation (%)										
Baseline	295	11·3	6·8–16·7	157	12·1	8·1–17·1	138	10·1	5·2–16·0	**0·029**
3rd month	259	16·3	11·5–23·8	129	14·5	10·5–19·0	130	20·1	12·5–28·5	**< 0·001**
Change from baseline		4·3	–0·6–9·8		2·1	–1·5–6·3		8·2	2·7–14·1	**< 0·001**
Hb (g/dl)										
Baseline	295	11·9	10·5–12·6	157	11·9	10·5–12·7	138	11·7	10·5–12·6	0·245
3rd month	259	12·4	11·5–13·2	129	12·2	11·7–13·1	130	12·6	11·5–13·2	0·229
Change from baseline		0·6	0·2–1·2		0·4	–0·1–1·1		0·9	0·3–1·3	**< 0·001**

IRT-only: received IRT alone; IRT-Pro: received IRT plus *L. plantarum 299v*; IQR: Interquartile range; TIBC: Total iron-binding capacity.

Mann–Whitney U test.

All relevant values were formatted in bold face.

At 3rd month of therapy, IRT-Pro (*n* 130) *v*. IRT-only (*n* 129) group had significantly higher serum levels for iron (76·0 (51·0–96·0) *v*. 60·0 (43·0–70·0) µg/dl, *P* < 0·001) and transferrin saturation (20·1 (12·5–28·5) *v*. 14·5 (10·5–19·0)%, *P* < 0·001) as well as higher change from baseline ferritin (13·0 (8·0–17·0) *v*. 5·0 (–1·0–15·0 ng/ml, *P* < 0·001), iron (23·5(5·0–48·0) *v*. 8·0 (–6·0–23·0) µg/dl, *P* < 0·001), transferrin saturation (8·2 (2·7–14·1) *v*. 2·1 (–1·5–6·3)%, *P* < 0·001) and Hb (0·9 (0·3–1·3) *v*. 0·4 (–0·1–1·1) g/dl, *P* < 0·001) (Table 3).

The 3^rd^-month TIBC levels were significantly lower (368·5 (327·0–402·0) *v*. 396·0 (374·0–421·0) µg/dl, *P* < 0·001) in the IRT-Pro group, as well as more remarkable decrease from baseline TIBC (–45·5 (–76·0/ −3·0) *v*. −11·0 (–40·0/24·0) µg/dl, *P* < 0·001) (Table 3).

### Patient demographics and baseline serum iron markers according to treatment discontinuation

No significant difference was noted in patients who discontinued treatment within 30 d and those who continued therapy in terms of patient demographics or baseline serum iron status markers (Table 4).

**Table 4.** Patient demographics and baseline serum iron status markers according to treatment discontinuation within 30 d (Mean values and sd; median values and minimum and maximum values)

	Treatment discontinuation within 30 d				
	Yes (*n* 30)		No (*n* 265)		
	Mean	sd	Mean	sd	*P* value
Patient demographics					
Age, Mean(sd)	35·2	10·1	36·3	10·7	0·611*
	*n*	%	*n*	%	
Gender, *n* (%)					
Male	1	3·3	10	3·8	0·690^†^
Female	29	96·7	255	96·2	
	Median	min-max	Median	min-max	
Baseline serum iron status markers, median(min-max)					
Ferritin (ng/ml)	7·0	2–36	6	1–36	0·116^‡^
Iron (Fe, µg/dl)	52·0	15–24	45·0	7–200	0·368^‡^
TIBC (µg/dl)	424·0	281–481	402·0	248–585	0·842^‡^
Transferrin sat (%)	13·20	3·4–27·1	11·2	1·3–42·3	0·354^‡^
Hb (g/dl)	11·5	9·1–13·7	11·9	6·5–14·9	0·344^‡^

IRT-only: received IRT alone; IRT-Pro: received IRT plus *L. plantarum 299v.*

*Independent *t* test, ^†^Fisher exact test, ^‡^Mann–Whitney U test.

## Discussion

Our findings revealed that at least one-third of patients developed gastrointestinal intolerance within 3 months of IRT, while the first 30 d of IRT was the most critical period for treatment discontinuation. Importantly, concomitant use of *L. plantarum 299v* probiotic supplementation during this critical period significantly reduced the likelihood of intolerance development (by ∼3 times) and treatment discontinuation (by∼5 times), increasing the gastrointestinal tolerability of the IRT, which also enabled the significantly improved serum iron status markers.

In addition, patients in the IRT-Pro group were more advantageous not only in terms of prevention of intolerance symptoms emerging over the course of IRT (loss of appetite, nausea, abdominal pain and constipation) but also in terms of amelioration of symptoms recorded at baseline (loss of appetite, nausea, abdominal pain and bloating), which seemed to positively affect their adherence to IRT. In contrast, IDA patients who received only IRT experienced significant increase of symptoms recorded at baseline such as loss of appetite, nausea and abdominal pain as well as a greater increase in constipation (∼10-fold *v*. ∼2-fold in IRT-Pro group). These findings seem notable given that constipation, nausea and abdominal pain were also the leading symptoms in patients who discontinued IRT within the first 30 d, and all were particularly noted in the IRT group.

Consistent with our findings, *L. plantarum 299v* supplementation has been reported to have many clinically confirmed positive effects such as improving gastrointestinal wellbeing in a healthy population, symptom relief by decreasing bloating and abdominal pain and normalisation of stool frequency as early as in the 2nd week of consumption in irritable bowel syndrome patients and decreasing the incidence of diarrhoea among patients receiving antibiotics^(20,21,33,34)^.

*L. plantarum 299v* has also the ability to survive passage through gastrointestinal tract and to inhibit the growth of potentially pathogenic bacteria in the intestine in addition to anti-inflammatory effects^(20,21,34)^. Notably, many studies indicated a link between gut microbiota (dysbiosis) and IDA as well as the association of iron therapy with the diversity and composition of the intestinal flora^(3,4,35–37)^.

While iron therapy is considered to normalise Hb within 2 months of treatment onset, and to build up iron stores within the next 2–3 months, many patients face considerable challenges in adhering to and persisting with the full iron replacement regimen^(38)^. Our results showed that IRT-Pro regimen had an NNT of 3, indicating that 3 patients have to be treated with IRT-Pro instead of IRT alone to prevent one additional treatment discontinuation Hence, use of *L. plantarum 299v* for the first 30 d of iron replacement seems to be a favourable treatment approach in IDA patients in terms of preventing the considerable gastrointestinal burden, including the amelioration of the symptoms already existent before IRT, and increasing patient adherence to IRT^(6,7,39)^.

The iron replacement aims not only to correct the Hb deficit but also to provide enough iron for measurable iron stores^(12)^. Our findings emphasise the potential benefit of using *L. plantarum 299v* supplementation in provision of more adequate supply of iron for Hb synthesis and in increasing the iron stores (improved iron status markers such as serum iron, ferritin, TIBC and transferrin saturation) and thus improving the effectiveness of oral iron replacement in patients with IDA.

The positive effects of using *L. plantarum 299v* supplementation for the first 30 d of IRT seem to indicate the likelihood of this probiotic strain to counteract the adverse effects of residual iron supplement that remains largely unabsorbed in the digestive tract, commonly causing adverse gastrointestinal events, reduced compliance and inefficient repletion of iron stores^(3,4,13,40)^.

In fact, given the improved tolerability and iron status markers within 3 months of therapy, use of *L. plantarum 299v* may also decrease the need for longer-term use of oral iron replacement or use of IV replacement, as well as the related gastrointestinal burden, offering a potentially cost-effective alternative in the management of IDA patients.

Data from clinical studies also revealed the association of *L. plantarum 299v* supplementation with increased bioavailability and absorption of iron in different types of iron deficiencies^(4,6,16,22,23,41)^. The exact mechanism behind the beneficial effects of *L. plantarum 299v* on dietary non-heme iron absorption is not known. Nonetheless, the process is considered likely to be mediated by the formation of bioavailable ferrous form by reduction of ferric iron (increasing iron uptake by enterocytes), the enhanced mucin production at the intestinal surface (promoting enterocyte iron uptake) and the immunomodulation promoting an anti-inflammatory immune response that suppresses the inflammatory cytokine-mediated increase in circulating hepcidin which otherwise blocks the passage of iron from the intestinal cell to the plasma (enhancing iron bioavailability)^(4,6,28,42)^. Hence, *L. plantarum 299v* supplementation seems to ensure adequate iron absorption by affecting multitude of factors implicated in the iron bioavailability, such as the choice of iron compound, the physiological state of the consumer (i.e. iron status, other nutritional deficiencies and inflammatory disorders) and the presence of enhancers and inhibitors of absorption in the food matrix^(42,43)^.

Similar to our results, in a recent randomised clinical trial in iron-deficient athletes, intake of *L. plantarum 299v* plus 20 mg of iron was considered likely to result in a more substantial and rapid improvement in iron status compared with 20 mg of iron alone^(44)^. In addition, *L. plantarum 299v* (plus sucrosomial iron and vitamin C) was reported to have a positive effect on the treatment and prevention of IDA, which causes higher iron blood levels (by 11 %) because of increased iron absorption compared with use of only sucrosomial iron and vitamin C^(7)^. Studies in pregnant women also showed the association of *L. plantarum 299v* with slower decline in maternal haematological and iron parameters across pregnancy in non-anaemic women as well as in those who are at risk for IDA in pregnancy^(28,29)^.

In a meta-analysis of eight studies on the effect of the probiotic *L. plantarum 299v* on iron absorption in healthy women of childbearing age, pregnant women and patients with IDA, *L. plantarum 299v* was concluded to significantly improve non-heme dietary iron absorption in humans^(6)^, while only one of eight studies reported improvement in iron status-related indices^(6,7)^. Importantly, providing data on the beneficial effects of *L. plantarum 299v* probiotic strain in IDA patients also in terms of iron status markers, our results indicate the likelihood of using *L. plantarum 299v* probiotic supplementation within the first 30 d of IRT to enable two sine qua non of the proper medication adherence and persistence, namely the perceived efficacy (reduced symptoms of iron deficiency) and the improved tolerability^(38,45)^. Nonetheless, there remains a need for further research toward filling gaps in the existing literature given that the effect of probiotics on body iron status remains to be less certain than their effects on iron absorption^(6)^.

Certain limitations to this study should be considered. First, single-centre study design, preponderance of female participants and exclusion of patients with known intolerance to oral iron or those with chronic diseases (i.e. İBS and inflammatory bowel disease) limit the generalisability of the findings to broader populations, including males, diverse ethnic groups and unselected patient populations. This might have also caused a selection bias toward a favourable tolerability for oral iron and affected our tolerability and treatment discontinuation results. Second, given the potential psychological effects of probiotic support, the lack of a placebo group seems to be another limitation of the present study in terms of the likelihood of a placebo effect with potential impact on the subjective symptom reporting. Nonetheless, the marked differences between treatment groups in gastrointestinal intolerance and treatment discontinuation seem to indicate a strong impact of probiotic therapy which cannot be explained solely by the placebo effect. Also, NNT analysis, which was performed particularly for this reason (lack of placebo arm), did not reveal a high NNT value which otherwise would indicate the likelihood of placebo effect. Third, assessment of symptom frequency was based on subjective reporting along with lack of items on symptom severity. Fourth, use of only the persistence (treatment discontinuation) measure of compliance with lack of adherence data is another important limitation of the study. Fifth, lack of data on iron regulation, including hepcidin, erythropoietin and erythroferrone, as the potential players, as well as the lack of data on purity testing of probiotic and no stool collection to demonstrate LP299V colonisation in gut or changes to microbiome are other limitations. Nevertheless, despite these certain limitations, given the restricted amount of data on iron status changes in IDA patients treated with probiotic plus IRT, our findings represent a valuable contribution to the literature.

### Conclusion

In conclusion, our findings in IDA patients revealed that using *L. plantarum 299v* probiotic supplementation during the first 30 d of IRT significantly reduced the gastrointestinal burden (particularly abdominal pain and bloating) related to IRT, the likelihood of developing de novo symptoms (loss of appetite, nausea, abdominal pain and constipation) under IRT and the likelihood of intolerance development (by ∼3 times) within 3 months of therapy and treatment discontinuation (by∼5 times) within 30 d of therapy. The improved gastrointestinal tolerability and patient adherence to oral IRT was also accompanied by a more remarkable improvement in serum iron markers in patients who received *L. plantarum 299v*. Hence, using *L. plantarum 299v* probiotic supplementation for the first 30 d of iron replacement seems to be a favourable treatment approach in IDA patients, given that oral IRT is limited by gastrointestinal side effects and noncompliance. Given the complex interplay between gut microbiota and iron bioavailability, and the research gap regarding the effects of probiotics on iron status, the long-term effects of different probiotic strains in combination with different iron preparations on iron status markers should further be investigated in unselected IDA populations.
